# Cortactin Is Required for Efficient FAK, Src and Abl Tyrosine Kinase Activation and Phosphorylation of *Helicobacter pylori* CagA

**DOI:** 10.3390/ijms22116045

**Published:** 2021-06-03

**Authors:** Jakob Knorr, Irshad Sharafutdinov, Florian Fiedler, Delara Soltan Esmaeili, Manfred Rohde, Klemens Rottner, Steffen Backert, Nicole Tegtmeyer

**Affiliations:** 1 Department of Biology, Division of Microbiology, Friedrich-Alexander University of Erlangen-Nuremberg, 91058 Erlangen, Germany; Jakob.Knorr@fau.de (J.K.); Irshad.Sharafutdinov@fau.de (I.S.); Florian.Fiedler@fau.de (F.F.); Delara.Esmaeili@fau.de (D.S.E.); steffen.backert@fau.de (S.B.); 2 Central Facility for Microscopy, Helmholtz Centre for Infection Research, 38124 Braunschweig, Germany; Manfred.Rohde@helmholtz-hzi.de; 3 Department of Cell Biology, Helmholtz Centre for Infection Research, 38124 Braunschweig, Germany; kro@helmholtz-hzi.de; 4 Division of Molecular Cell Biology, Zoological Institute, Technische Universität Braunschweig, 38106 Braunschweig, Germany

**Keywords:** Abl, *Helicobacter*, cancer, cortactin, FAK, pathogenesis, pathogenicity island, signaling, Src, virulence

## Abstract

Cortactin is a well-known regulatory protein of the host actin cytoskeleton and represents an attractive target of microbial pathogens like *Helicobacter pylori*. *H. pylori* manipulates cortactin’s phosphorylation status by type-IV secretion-dependent injection of its virulence protein CagA. Multiple host tyrosine kinases, like FAK, Src, and Abl, are activated during infection, but the pathway(s) involved is (are) not yet fully established. Among them, Src and Abl target CagA and stimulate tyrosine phosphorylation of the latter at its EPIYA-motifs. To investigate the role of cortactin in more detail, we generated a CRISPR/Cas9 knockout of cortactin in AGS gastric epithelial cells. Surprisingly, we found that FAK, Src, and Abl kinase activities were dramatically downregulated associated with widely diminished CagA phosphorylation in cortactin knockout cells compared to the parental control. Together, we report here a yet unrecognized cortactin-dependent signaling pathway involving FAK, Src, and Abl activation, and controlling efficient phosphorylation of injected CagA during infection. Thus, the cortactin status could serve as a potential new biomarker of gastric cancer development.

## 1. Introduction

*Helicobacter pylori* usually infects its human hosts during early childhood and is one of the most successful pathogens in history, with approximately 50% of the global population colonized. In most *H. pylori*-positive humans, the infection proceeds asymptomatic. However, the pathogen is also known as a causative agent of multiple gastric diseases, such as severe gastritis, peptic ulceration, or stomach cancer in a subset of patients [1,2,3]. The mechanisms by which *H. pylori* causes gastric cancer have been under investigation ever since the link between disease and pathogen was discovered [4], but can now best be described as two major pathways: One comprises the direct impact on host cell signaling via *H. pylori’s* virulence factors, which leads to altered cell behavior and malignant cellular autonomy. The second pathway is indirect and occurs as a side effect of the chronic inflammatory response in infected tissue caused by long-term infection, resulting in increased cell turnover and accumulation of mitogenic defects [5,6,7]. Enabling a persistent, long-term infection is a crucial process for the bacterium, requiring a multitude of steps such as survival in the harsh conditions of the human stomach, reaching the gastric epithelial cell layer and adhering to it, followed by manipulating host cell signaling to the pathogen’s advantage [8]. For example, *H. pylori* senses differences in pH gradients within the gastric environment, allowing it to move through the gastric mucus layer in order to reach epithelial cells [9,10]. *H. pylori* is also capable of lowering stomach acidity around its microenvironment by the secretion of a urease enzyme complex [11]. In spite of all this knowledge, the precise mechanisms involving chronic colonization by *H. pylori* are still not fully elucidated.

*H. pylori* attaches to gastric epithelial cells using various outer-membrane adhesins, including BabA/B, SabA or HopQ, and delivers multiple virulence factors, such as the lipopolysaccharide metabolite ADP-heptose, the toxin VacA, or effector proteins into the host [3,5,6,12,13,14]. Other virulence factors are secreted into the supernatant, like the serine protease HtrA, which functions to weaken epithelial cell-to-cell-junctions by cleavage of the tumor suppressor E-cadherin and other proteins [15,16]. This allows *H. pylori* to gain access to the intercellular space and subsequently basolateral membranes of epithelial cells [17]. One of the best-studied virulence factors in this scenario is the translocated effector protein CagA (encoded by the cytotoxin-associated gene A). The *cagA* gene is located in the so-called *cag* pathogenicity island (*cag*PAI), an approximately 40 kb stretch of DNA, which harbors about 30 different genes and represents a strong virulence marker [18]. This *cag*PAI forms a type IV secretion system (T4SS) pilus, which is utilized to deliver CagA, chromosomal DNA, and ADP-heptose into the host target cells [3,14,19,20]. Inside the host cell, CagA becomes tyrosine-phosphorylated at the so-called EPIYA-motifs by members of the Src and Abl kinase families [21,22,23]. CagA can then interact with a plethora (>25) of different host cell proteins, both in a phosphorylation-dependent as well as phosphorylation-independent manner, in order to influence host cell signaling [24,25] including pathways promoting carcinogenesis [26,27].

The actin-regulatory protein cortactin, encoded by the *cttn* gene, is one of the factors used by *H. pylori* to manipulate host cell signaling [28]. Cortactin can be detected as two bands of approximately 80 and 85 kDa, called p80 and p85 when analyzed by SDS-PAGE [29,30]. The protein consists of an acidic domain at its N-terminus, a more central F-actin binding domain, a proline-rich region, and a Src-homology 3 (SH3) domain [31,32]. In healthy tissue, cortactin presumably acts by binding to actin filaments (F-actin) and to the prominent actin nucleation factor Arp2/3 complex, the latter of which generates branches within actin filament networks [33,34]. Cortactin may stabilize Arp2/3 complex-containing branches and thereby operate as an antagonist of de-branching factors such as members of the coronin family [35,36]. Due to these important functions, cortactin has also been described as the “Achilles heel” of the cytoskeleton since being exploited by multiple pathogens [37]. 

*H. pylori* specifically manipulates the phosphorylation status of cortactin by inactivation of Src, via a negative feedback loop mechanism, induced by phosphorylated CagA and carboxy-terminal Src kinase (Csk), as well as tyrosine dephosphorylation by an hit-herto unknown phosphatase [38,39]. In addition, *H. pylori* stimulates the activation of MAP kinases Erk1/2 to induce cortactin phosphorylation at serines 405 and/or 418 [40]. Tyrosine-dephosphorylated and serine-phosphorylated cortactin is then able to bind to focal adhesion kinase (FAK) [41]. In this way, it appears that *H. pylori* may control FAK activity and cell adhesion in the gastric epithelium. However, studies regarding the impact of cortactin during infection with *H. pylori* have mostly relied on in vitro experiments using knockdown of cortactin e.g., by RNA interference [41,42,43]. To investigate cortactin’s precise function during *H. pylori* infection in more detail, we here employed CRISPR/Cas9-mediated genome editing to generate stable and complete Cortactin-deficient AGS cell lines (AGSΔ*cttn*) subjected to infection experiments. Using this approach, we discovered that cortactin is required for profound activation of the non-receptor tyrosine kinases FAK, Src, and Abl, followed by efficient phosphorylation of injected CagA upon infection.

## 2. Results

### 2.1. Cortactin Knockout Does Not Affect Expression of Other Cytoskeletal Proteins, but Increases Actin Stress Fiber Formation and Cell Area

**Figure 1:**
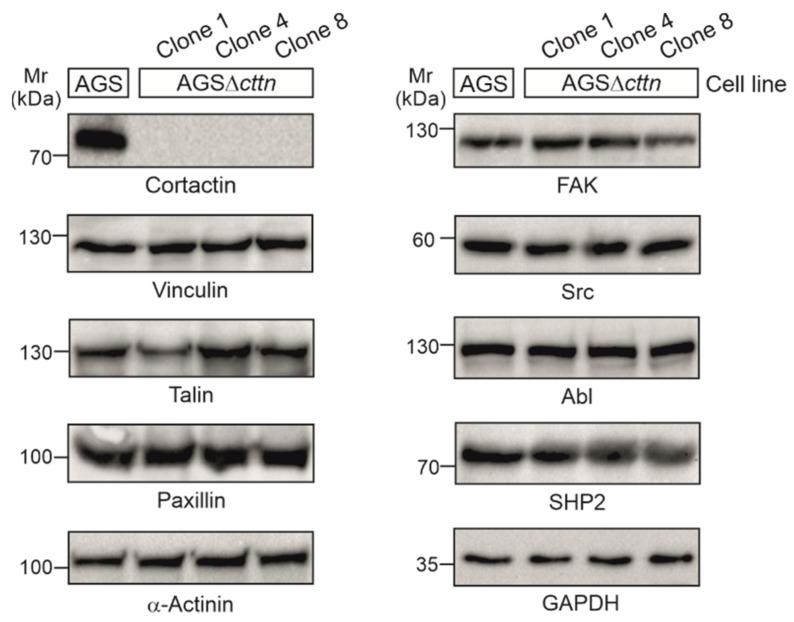
Cortactin was knocked-out in three clones of AGS cells using the CRISPR/Cas9 system. Cortactin expression could not be detected in the AGSΔ*cttn* clones 1, 4, and 8, while expression of other proteins (vinculin, talin, paxillin, α-actinin, FAK, Src, Abl, and SHP2) involved in signaling to actin remodeling at the plasma membrane and in anchoring cells to the extracellular matrix through focal adhesions remained unchanged. GAPDH was used as a loading control.

Using CRISPR/Cas9, we generated three cortactin knockout clones in AGS cells, designated clone 1, 4, and 8, respectively. Cortactin knockout was confirmed via Western blotting (Figure 1). The expression of other prominent cytoskeletal and signaling factors in cortactin knockout clones, including vinculin, talin, paxillin, α-actinin, FAK, Src, Abl, and SHP2, were similar to wild-type (wt), suggesting that cortactin removal did not affect the translation of all these proteins (Figure 1). 

**Figure 2:**
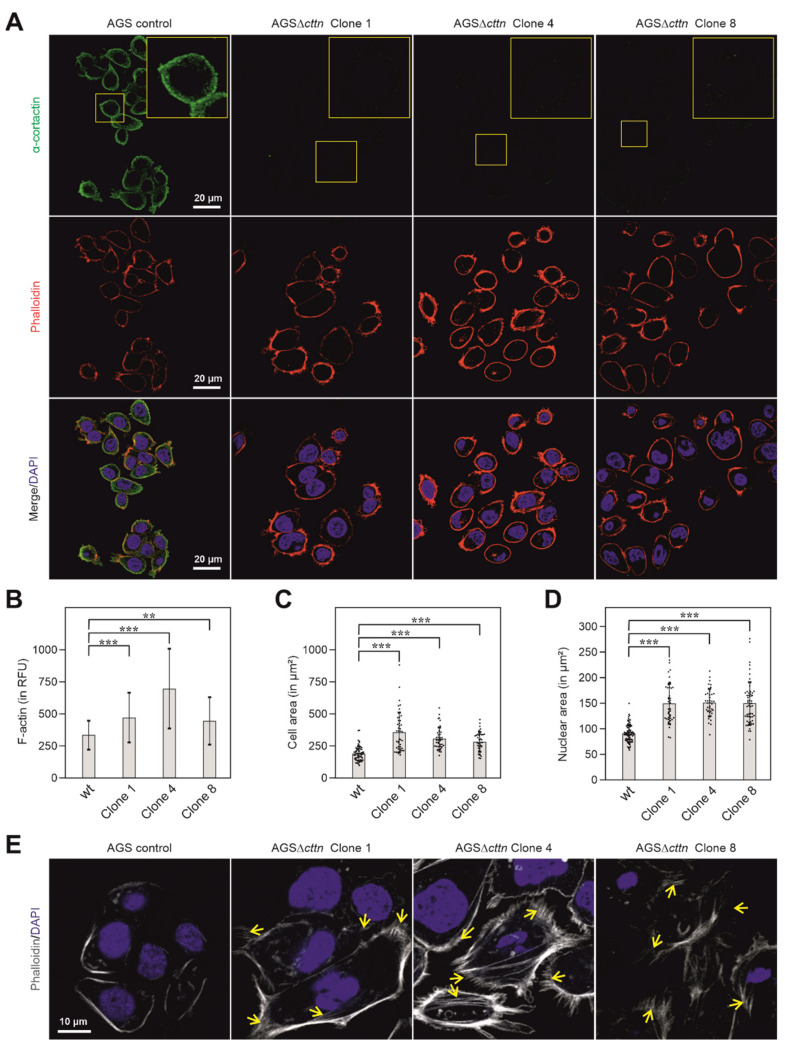
Cortactin (green) distribution in AGS cells counterstained with phalloidin (actin filaments, red) and DAPI (blue, nuclei) as assessed by CLSM analysis. (**A**) In AGS wt cells, cortactin prominently accumulated at the cell periphery and in spot-like structures in the cytoplasm, whereas no such staining was obtained in AGSΔ*cttn* cells (clones 1, 4 and 8). (**B**) Bars depicting mean ± SD of fluorescence intensity (total cell fluorescence divided by cell area) denote marked differences in cellular F-actin contexts between AGSΔ*cttn* and wt cells; RFU—relative fluorescence units. (**C**) Scatter plot with bars depicting mean ± SD of cell areas, revealing that AGSΔ*cttn* cells display larger average cell areas than AGS wt cells. Each dot represents measurement of a single cell. (**D**) Scatter plot with bars depicting quantifications of nuclear areas (means ± SD) in different AGS cell clones, as indicated. Each dot represents measurement of a single nucleus. Nuclei were stained with DAPI. (**E**) CLSM of basal layers of AGS cells reveal extensive stress fibers formation (yellow arrows) in AGSΔ*cttn* cells. Statistical differences are indicated by asterisks; *** correspond to *p* ≤ 0.001 and ** correspond to *p* ≤ 0.01.

To further verify the knockout of cortactin in the three AGSΔ*cttn* clones, confocal laser scanning microscopy (CLSM) was performed. Using cortactin-specific antibodies, the immunofluorescence staining experiments confirmed the disruption of the cortactin gene in all three AGSΔ*cttn* clones (Figure 2A). AGSΔ*cttn* furthermore exhibited significantly increased F-actin fluorescence signal compared to AGS parental control cells (Figure 2B). Remarkably, we also observed that all AGSΔ*cttn* clones exhibit enlarged spreading areas in comparison to AGS control cells (Figure 2C). Quantification of cell areas showed that the mean ± SD value of AGS control cells was 191 ± 57 µm^2^, while for AGSΔ*cttn* clones 1, 4, and 8, average areas of 358 ± 154 µm^2^, 308 ± 86 µm^2^, and 285 ± 75 µm^2^ were measured, respectively (Figure 2C). The nuclei of cortactin-deficient AGS cells were also significantly enlarged (150 ± 39, 151 ± 27, 151 ± 41 µm^2^ in clones 1, 4 and 8, respectively) versus 91 ± 17 µm^2^ in AGS wt cells (Figure 2D). Alongside with the enlarged cellular area, AGSΔ*cttn* clones also exhibited augmented actin stress fiber formation as compared to AGS control cells (Figure 2E, highlighted by arrows). Altogether, immunofluorescence microscopy analysis showed that cortactin gene knockout in AGS epithelial cells was achieved and is associated with the (i) increase of cellular and nuclear areas as well as (ii) enhancement of stress fiber formation.

### 2.2. Efficient Phosphorylation of CagA Is Diminished in AGSΔcttn Cells

**Figure 3:**
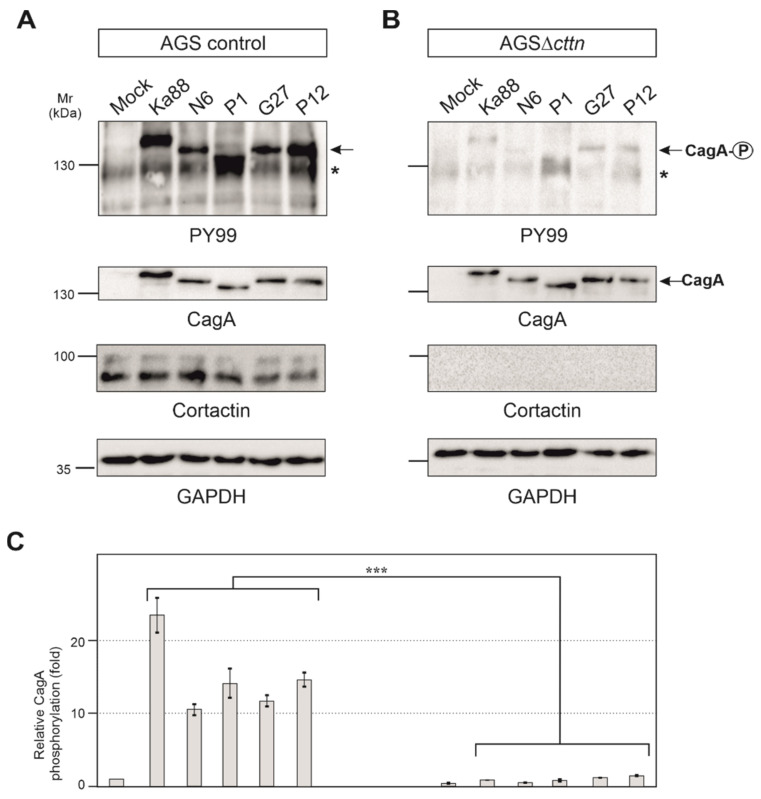
AGS wt and AGSΔ*cttn* cells were infected for 6 h with five different strains of *H. pylori*. (**A**,**B**) Tyrosine phosphorylation of CagA was dramatically reduced in AGSΔ*cttn* cells in all infections as seen using PY99 antibody. Phospho-CagA is marked with arrows. The asterisks denote an unknown phosphorylated host cell protein. A general CagA antibody was used to confirm that cells were infected with equal amounts of bacteria. The cortactin blot confirmed the knockout of the protein. GAPDH was used as loading control. (**C**) Statistical analysis confirms the significant (*** corresponding to *p* ≤ 0.001) reduction of CagA phosphorylation compared to background signal. Infections in AGS wt showed a 23-fold increase of CagA phosphorylation in *H. pylori* strain Ka88, and the remaining strains exhibited a 10- to 15-fold increase. During infection, virtually no increase in CagA phosphorylation (compared to background) could be detected in AGSΔ*cttn* cells. Representative data are shown for AGSΔ*cttn* clone 1.

AGS control and AGSΔ*cttn* cells were then infected under identical conditions with five different *H. pylori* T4SS-positive wt strains (Ka88, N6, P1, G27 and P12). Strong phosphorylation of injected CagA was detected in AGS control cells employing the pan-tyrosine antibody PY99 (Figure 3A). Phosphorylation of CagA in AGSΔ*cttn* cells, however, was drastically reduced in comparison to AGS control cells (Figure 3B). Diminished phosphorylation of CagA in AGS∆*cttn* cells was further confirmed by statistical analysis of the blots, which confirmed a high level of significance (*p* ≤ 0.001). Additionally, CagA phosphorylation levels of all five *H. pylori* strains were reduced to similar extents in all AGS∆*cttn* cell clones, suggesting that the difference derives from the absence of cortactin and is independent from translocated CagA variant (Figure 3C and data not shown). 

### 2.3. Bacterial Binding to Cells and T4SS Pilus Formation Are Not Affected by Cortactin Knockout

**Figure 4:**
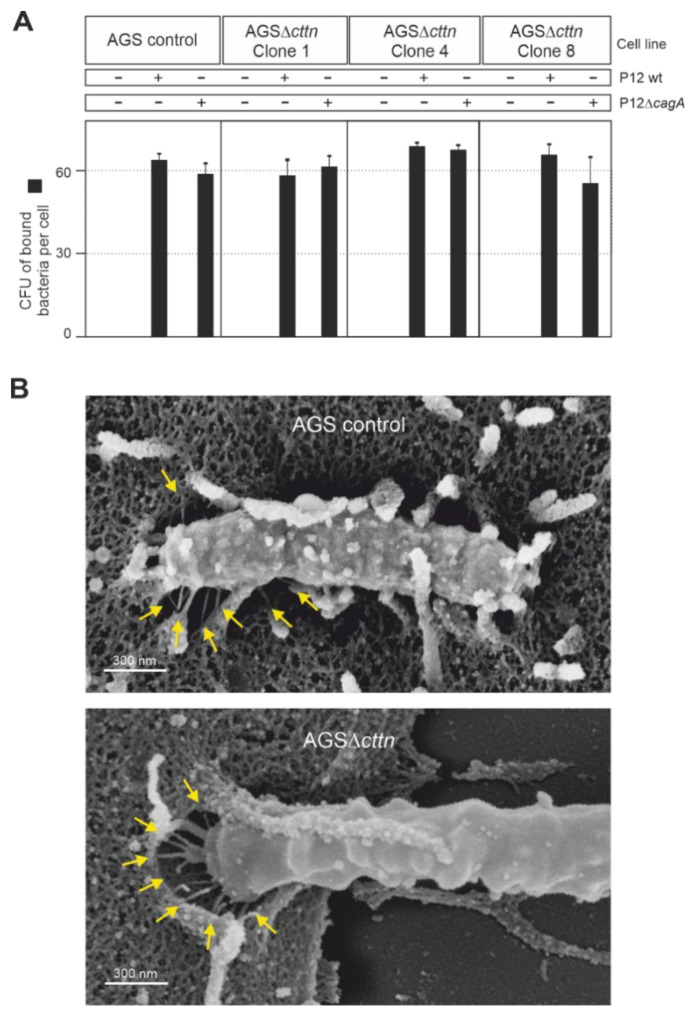
AGS wt and AGSΔ*cttn* were infected for 6 h with *H. pylori* strain P12 and its isogenic mutant, P12Δ*cagA* used as a control. (**A**) The number of *H. pylori* bound per cell was similar in all infections, regardless of bacterial strain or cell line used. (**B**) Scanning electron micrographs showing that in both AGS wt and AGSΔ*cttn* infections with *H. pylori*, the formation of T4SS pili (arrows) was not compromised. Representative data are shown for AGSΔ*cttn* clone 1.

As cortactin is readily observed in actin structures at the plasma membrane [29], we next asked if the downregulation of CagA phosphorylation in AGS∆*cttn* cells may be due to impaired bacterial binding to the cells. To address this option, we infected AGS control and AGS∆*cttn* cells with wt *H. pylori* versu*s* isogenic Δ*cagA* mutant as control at an MOI of 100, followed by quantification of the bacteria bound. The results show that all bacterial strains bound to both cell types with high efficiency of roughly 60 bacteria per cell (Figure 4A). Some minor differences were seen in bacterial attachment, but these were not statistically significant. We then asked if the T4SS is functional in the cortactin knockout cells. For this purpose, we infected AGS control and AGS∆*cttn* cells with wt *H. pylori* and investigated T4SS pilus formation. Scanning electron microscopy revealed both intimate binding of *H. pylori* to the cell surface and proper T4SS pilus formation in both cell types (Figure 4B, arrows). Together, these control experiments verify that the lack of CagA phosphorylation is not due to impaired bacterial cell binding or a T4SS defect in infected AGS∆*cttn* cells. 

### 2.4. Cortactin Is Necessary for Effective Activation of the Kinases FAK, Src and Abl by H. pylori

**Figure 5:**
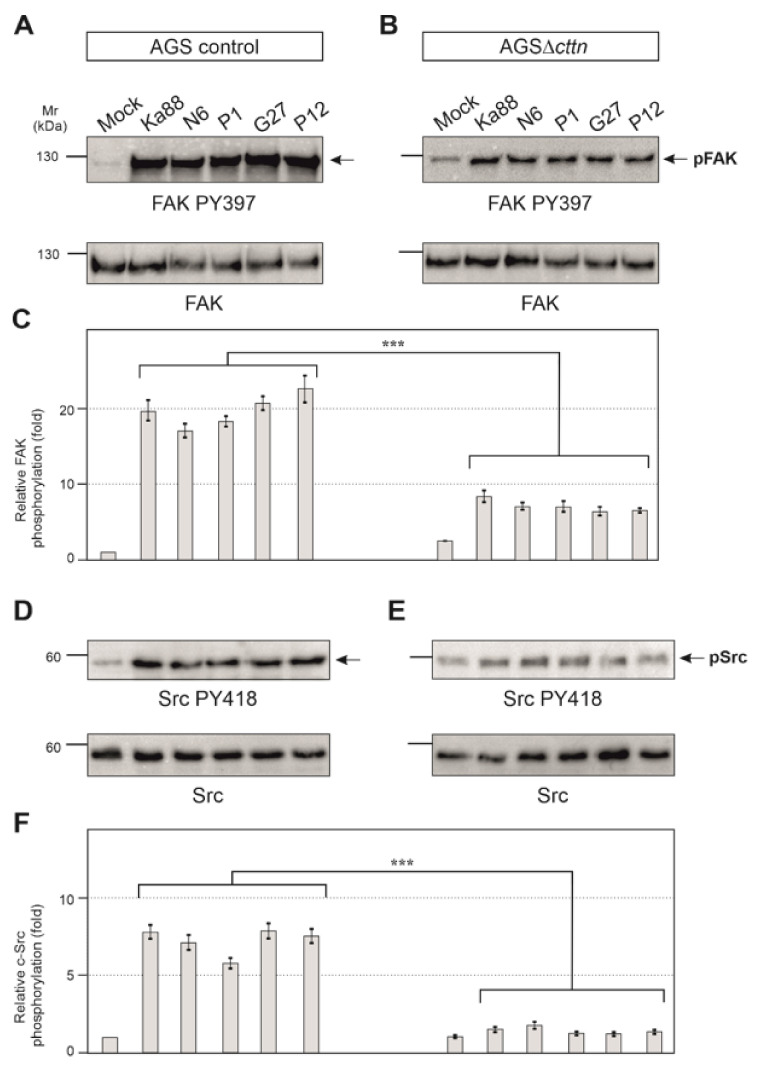
Infection experiments of AGS wt and AGSΔ*cttn* cell lines with five different strains of *H. pylori* for 6 h. (**A**) Phosphorylation of focal adhesion kinase (FAK) at tyrosine 397 increased over the course of the infection in AGS wt cells. (**B**) Phosphorylation of FAK Y397 post-infection in AGSΔ*cttn* was only slightly enhanced as compared to uninfected cells. (**C**) Statistical analysis revealed an approximately 20-fold increase in infected AGS wt cells compared to uninfected cells, whereas AGSΔ*cttn* cells showed an approximately 8-fold increase. Infected and uninfected AGSΔ*cttn* cells showed more or less the same level of FAK PY397 within each cell line regardless of *H. pylori* strain. (**D**) Phosphorylation of Src at tyrosine 418 increased over the course of infection of AGS wt cells. (**E**) Phosphorylation of Src PY418 was hardly detectable in AGSΔ*cttn* cells during *H. pylori* infection. **(F**) Statistical analysis revealed a 5- to 7-fold increase in infected AGS wt cells compared to uninfected cells, while AGSΔ*cttn* showed only a 2-fold increase or less. Representative data are shown for AGSΔ*cttn* clone 1. Differences were confirmed to be statistically significant; *** *p* ≤ 0.001.

Next, we aimed to investigate if the tyrosine kinases of CagA in AGS∆*cttn* cells can be properly activated by *H. pylori*. To answer this question, we infected AGS control and AGS∆*cttn* cells with the same five *H. pylori* wt strains used above and monitored tyrosine kinase activity using activation-specific phospho-antibodies. We have previously shown that cortactin can promote FAK phosphorylation at tyrosine residue 397 in its active center upon their interaction [41]. As expected for AGS control cells, the results show that infection leads to an approximately 20 ± 3 fold increase of FAK activity by all *H. pylori* strains as compared to the uninfected mock control (Figure 5A). In contrast, FAK activation in infected AGSΔ*cttn* cells under the same conditions was strongly reduced (Figure 5B). Statistical analysis of relative FAK phosphorylation confirmed a highly significant (*p* ≤ 0.001) 2- to 3.5-fold decrease of FAK phosphorylation in AGSΔ*cttn* cells compared to their parental control (Figure 5C). 

**Figure 6:**
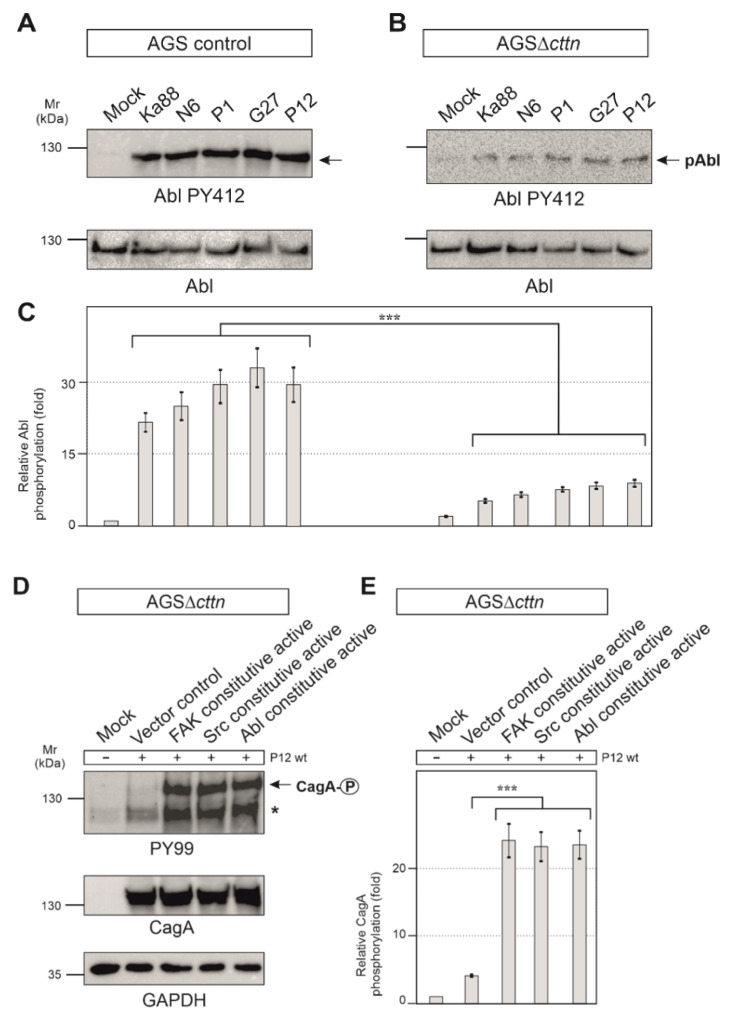
(**A**) Infections of AGS wt cells with five different strains of *H. pylori* for 6 h. Phosphorylation of Abl at tyrosine 412 increased over the course of infections with all *H. pylori* strains. (**B**) For infections of AGSΔ*cttn* cells with five different strains of *H. pylori* for 6 h, only a slight activation of Abl was observable. (**C**) Statistical analysis revealed a 22- (Ka88) to 32- (G27) fold increase in infected AGS wt cells compared to uninfected cells. However, AGSΔ*cttn* cells exhibited only a 5- to 9-fold increase of Abl PY412. *** *p* < 0.001. (**D**,**E**) AGSΔ*cttn* cells were transfected with constitutively active variants of FAK, Src, and Abl, followed by infection with *H. pylori* strain P12. Transfections with these mutants rescued the phosphorylation phenotype of the bacterial effector protein CagA. Phosphorylation of CagA increased by a factor of about 23 in all transfections with active kinases. Representative data are shown for AGSΔ*cttn* clone 1. The single asterisk denotes an unknown phosphorylated host cell protein. *** *p* ≤ 0.001.

It is well described that CagA phosphorylation in host cells is controlled by Src and Abl tyrosine kinases [23]. Next, we therefore monitored the activity of Src and Abl. Src activity was measured with the activation-specific Src-PY418 antibody. AGS control cells displayed Src activation following infection with the five different *H. pylori* strains in a range of roughly 5- to 7-fold, whereas in AGSΔ*cttn* cells, Src activation was found to be 2-fold or less (Figure 5D,E). Statistical analysis confirmed this observation and showed a highly significant (*p* ≤ 0.001) decrease in Src phosphorylation in AGSΔ*cttn* cells compared to wt cells (Figure 5F). The kinase Abl was also activated following *H. pylori* infection in AGS control cells, as shown using an Abl PY412 antibody. Activation of Abl in AGS control cells varied between 22- to 32-fold among the different *H. pylori* strains, however, AGS∆*cttn* cells exhibited only a 5- to 9-fold increase (Figure 6A,B). The decrease of Abl PY412 staining in AGS∆*cttn* cells compared to controls was again confirmed to be statistically significant (*p* ≤ 0.001) (Figure 6C). Taken together, all these data suggest that genetic cortactin removal is associated with a remarkable deficiency in activating the non-receptor tyrosine kinases FAK, Src, and Abl. 

### 2.5. Constitutively Active FAK, Src or Abl Rescue CagA Phosphorylation in AGSΔcttn Cells

Aforementioned observations led us to hypothesize that cortactin expression is required in gastric epithelial cells to promote a signaling pathway to activate various tyrosine kinases that are important for phosphorylation of injected CagA. To investigate if this idea is correct, we asked if we were able to rescue the CagA phosphorylation defect by ectopic expression of constitutively active constructs of FAK, Src, or Abl, respectively. For this purpose, we first utilized a phosphorylation-mimetic FAK mutation construct, in which the autophosphorylation site at tyrosine 397 was exchanged for glutamic acid to generate the active form of FAK [44]. Secondly, Src was mutated at position 527 from tyrosine to phenylalanine, which impaired the intramolecular autoinhibitory binding of its SH2 domain, prohibiting inactivation of the kinase [45]. Lastly, two point-mutations in Abl exchanging proline for glutamic acid in the SH2-linker-domain at positions 242 and 249 resulted in a permanently activated state of the kinase [46]. AGSΔ*cttn* cells were transiently transfected for 48 h with empty control vector or plasmids encoding respective constitutively active variants of FAK, Src, or Abl, followed by infection with *H. pylori*. Transfection of AGSΔ*cttn* cells with any of the constitutively active kinase variants was sufficient for rescuing the phosphorylation of CagA to almost wt levels, while transfection with the empty vector control did not (Figure 6D). Relative CagA phosphorylation levels in transfections with all mentioned kinase variants were highly similar to each other, and the difference in each case to vector control confirmed to be highly statistically significant (*p* ≤ 0.001) (Figure 6E). 

## 3. Discussion

Various groups have created cortactin knockout mouse models and commonly reported comparably mild phenotypes at least in non-challenging conditions, such as enhanced vascular permeability [47] or subtle changes in dendritic spine morphology and dynamics [48]. One group reported lethality caused by cortactin deletion in very early embryonic stages [49], but this phenotype was likely caused by the specific gene trapping method used, as implied by the existence of homozygous null animals in more recent work (see e.g., [47]). Apart from the mice, multiple cell lines of varying tissues were also produced, most notably embryonic fibroblasts, which again exhibited comparably modest impairment of cell migration efficiency and signaling defects to small GTPase activation for instance. Aside from that, however, these cell lines were comparable to control their growth rates and morphology [50,51]. Cortactin is a prominent regulatory protein of the actin cytoskeleton and was proposed to play important roles in actin remodeling by means of its interaction with actin filaments and Arp2/3 complex [31]. Furthermore, by binding specific proteins via its SH3 domain, cortactin is able to influence cellular behavior, such as cell migration by interacting with N-WASP [52], cell-to-cell adhesion through the tight junction protein ZO-1 [53], or endocytosis via dynamin [54]. These remarkable attributes have made cortactin an attractive target of numerous microbial pathogens, to manipulate the host cell cytoskeleton to their own benefit [37]. One of the pathogenic bacteria to exploit cortactin is the gastric pathogen *H. pylori* [41]. Here we discovered a novel signaling pathway, in which *H. pylori* manipulates cortactin to induce the phosphorylation of its effector protein CagA.

Although previous studies have attempted to investigate the importance of cortactin in gastric epithelial cells, no complete knockout has been generated in these studies. Instead, siRNA or similar methods were applied to achieve an incomplete knockdown, which offers only limited insight into the precise mechanisms of cortactin-dependent signaling [41,55]. Here, we generated the first stable knockout of cortactin in a gastric epithelial cell line through application of the CRISPR/Cas9 method. As we could show, deletion of cortactin in AGS cells had no noticeable effect on the rate of expression of other proteins found in nascent protrusions or adhesions, like vinculin, talin, paxillin, or FAK. Immunolabeling experiments showed two important phenotypic differences between wt and cortactin-deficient AGS cells. Deletion of the cortactin gene led to the accumulation of actin stress fibers as well as enlargement of the spreading area of AGS cells. These two features indicate a critical function for cortactin not only in cell scattering or migration, as reported previously [41,56], but also in maintaining cellular structure (this study). Similar to AGSΔ*cttn* cells, wild-type MDA-MB-231 breast carcinoma cells displayed a large, flat shape with the actin cytoskeleton primarily organized as stress fibers [57]. Upon cortactin activation through insulin-like growth factor-1 (IGF-1) or EGF treatment in MDA-MB-231 wt cells, however, these stress fibers reorganized into membrane ruffles and lamellipodia, leading to the assumption that cortactin activity was required for cytoskeletal remodeling [57]. Consistently, PDGF-mediated induction of membrane ruffling and lamellipodia formation was not only less prominent in the absence of cortactin as compared to controls, but also coincided much less upon cortactin deficiency with the dissolution of stress fibers and in particular, the focal adhesions anchoring them [51]. Why and how the observed increase in stress fibers coincides with enhanced cell areas in our cortactin-deficient AGS cells, however, remains not entirely clear. It is worth noting though that the absence of rapid actin remodeling at the cell periphery by removal of lamellipodia, caused for instance by genetic removal of Rac1 GTPase, does also not reduce cell spreading efficiency [58]. In addition, the induced inhibition of Arp3 expression, an essential subunit of the Arp2/3 complex mediating actin filament branching in lamellipodia, not only increased rather than reduced cell spreading, but also dramatically increased cell and nuclear sizes [59]. All these data show that parameters such as cell size and spreading area can be completely uncoupled from dynamic actin remodeling in lamellipodia, presumably promoted by Arp2/3 complex activity and its binding and tuning factors including cortactin. Finally, deletion of other actin-binding proteins such as cofilin-1 and actin-depolymerizing factor (ADF) again resulted in comparable phenotypic characteristics in skin keratinocytes, including accumulation of contractile actin stress fibers [60]. This might suggest that inhibition of F-actin turnover, as for instance caused by cortactin or ADF/cofilin family member removal, may indeed promote the formation of more stable, contractile actin structures, such as stress fibers. 

The molecular basis behind our observations might be connected to insufficient FAK activation in AGSΔ*cttn* cells, causing a switch from FAK-dependent to FAK-independent integrin signaling, similar to the sequence of events observed in FAK-deficient fibroblasts, which display huge morphological and migratory defects and again highly increased stress fibers and focal adhesions [44]. Deletion of the integrin β1 receptor, a FAK binding protein, was also reported to increase cell spreading area and the number of focal adhesions in human breast cancer cells, while deletion of integrin β3 had no influence on either parameter [61]. Rho GTPase signaling pathways seem to be likely targets, as activation of RhoA is negatively regulated by activated FAK [62], and increased activities of RhoA and its target ROCK1 have been observed in cortactin knockdown Caco2 cells [55]. In contrast, actin stress fiber formation in muscle cells was shown to be completely inhibited upon cortactin knockdown by siRNA [63]. Thus, it seems possible that cortactin contributes differentially to stress fiber formation depending on cell type and condition. Whatever the case, future research will have to clarify all these questions.

**Figure 7:**
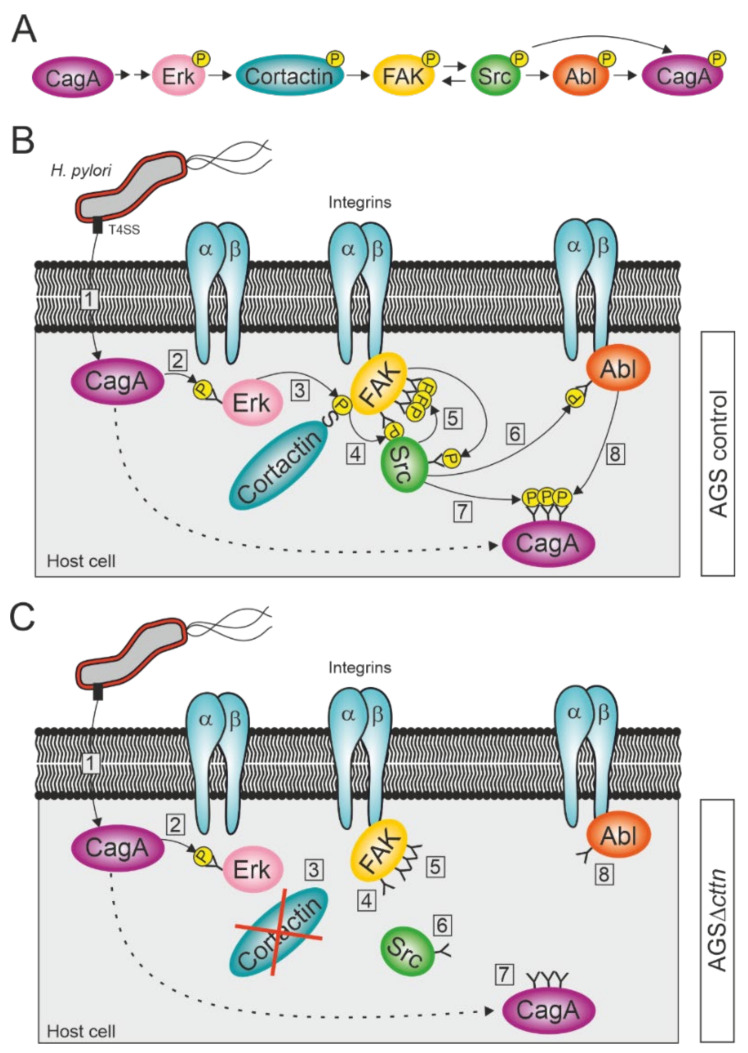
Proposed model for the activation of FAK, Src, and Abl kinases during infection with *H. pylori*. (**A**) Schematic overview of successive activation of host cell proteins, resulting in CagA phosphorylation. (**B**) *H. pylori* attaches to the membrane of AGS wt cells and injects CagA into the cytoplasm via its T4SS (step 1). CagA induces the activation of the serine kinase Erk in a phosphorylation-independent fashion (2). Erk phosphorylates cortactin at serine 405, allowing an interaction between cortactin and integrin-bound FAK through the SH3 domain of cortactin (3). The binding of cortactin to FAK induces autophosphorylation of FAK at tyrosine 397 (4). Src binds to FAK PY397 and becomes phosphorylated at tyrosine 418 by FAK, while also phosphorylating the remaining tyrosine phosphosites of FAK (5). Activated Src phosphorylates integrin-bound Abl at tyrosine 412 (6). Activated Src and Abl then phosphorylate CagA at its EPIYA-motifs (7 and 8). (**C**) *H. pylori* attaches to the membrane of an AGS∆*cttn* cell and injects CagA into the cytoplasm via T4SS (1), where it induces the activation of Erk in a phosphorylation-independent fashion (2). Due to the absence of cortactin, the signaling pathway, however, comes to a premature end after Erk activation (3), since cortactin cannot induce anymore the autophosphorylation of FAK (4). Thus, Src does not bind to FAK and therefore neither phosphorylates FAK’s remaining phosphosites nor becomes phosphorylated itself (5). As Src is still in its inactive form, it also fails to activate Abl (6), which therefore results in diminished phosphorylation of CagA (steps 7 and 8).

Our observation that phosphorylation of CagA injected by *H. pylori* is widely diminished in AGS cells lacking cortactin expression was very surprising. We therefore investigated the phosphorylation status of the three kinases FAK, Src, and Abl before and after infection with *H. pylori*. The bacterium injects CagA into the host cell via a T4SS, where it induces Erk1/2 MAP kinase activation, as has been previously established [64,65]. Erk1/2 activation is achieved through activation of the Ras/MEK/Erk signaling pathway by CagA interacting with Grb2 and does not require CagA phosphorylation [65,66,67]. Erk1/2 activation can be further enhanced by phosphorylated CagA interacting with the phosphatase SHP2 [68]. Activated Erk1/2 in turn can phosphorylate cortactin at serines 405 and 418 [40], which allow cortactin to bind to FAK and induce its autophosphorylation [41]. This enables the binding of Src kinase to FAK and phosphorylation of the remaining phospho-tyrosine sites on FAK to fully activate the kinase [41,69,70]. Fully activated FAK in turn phosphorylates Src, as has already been shown in neuroblastoma cells [71]. Activated Src is then able to phosphorylate and activate Abl [72]. This is in agreement with our results, which showed that while autophosphorylation of FAK at tyrosine 397 still occurred in AGSΔ*cttn* during a *H. pylori* infection, the rate of activation was reduced by more than 50% compared to control cells. Similarly, activation of Abl was also reduced, though still observable, whereas activation of Src was barely detectable. Both Src and Abl are known to phosphorylate CagA at its EPIYA-motifs [20,21,22,23,24,73], explaining the reduced phosphorylation of CagA we observed. Interestingly, transfection of AGS∆*cttn* cells with constitutively active mutants of either FAK, Src, or Abl rescued the phosphorylation status of CagA at approximately the same level for all three kinases as seen in infected AGS wt cells. Taken together, these results suggest a single signaling pathway, with cortactin upstream of the three kinases. We therefore propose that CagA induces its own tyrosine phosphorylation via cortactin-dependent activation of FAK, Src, and Abl (Figure 7A–C).

Both Src and Abl often exhibit increased activity in different tumor cells [74,75], so it comes as no surprise that they get exploited to target CagA, one of the virulence factors most closely associated with *H. pylori*’s ability to cause gastric cancer [76]. Aside from the pathway proposed above, the diminished signal of phosphorylated CagA could arise from other defects, such as reduced adherence of *H. pylori* to the host cell, or diminished formation of T4SS pili to inject CagA. However, neither attachment efficiency of the bacteria nor their adherence as observed by electron microscopy were reduced in AGS∆*cttn* cells as compared to cortactin-expressing control cells, and so failed to provide an explanation for the defects observed. Likewise, the electron microscopy confirmed that the formation of T4SS pili used for CagA injection was also not hampered in AGS∆*cttn* cells. Moreover, Western blots revealed comparable levels of CagA in both AGS wt and cortactin knockout cell lines, disproving the idea that a reduction of intracellular CagA would be able to explain reduced CagA phosphorylation levels. 

Finally, cortactin has been described as a potential biomarker in various adenocarcinoma and indicates a poor survival prognosis for patients [77,78]. By comparison, the development of gastric cancer is closely associated with persistent CagA-positive *H. pylori* infections [76,79,80], and injected and phosphorylated CagA is defined as the first bacterial oncoprotein [7]. Therefore, it is remarkable that the phosphorylation level of intracellular CagA was dramatically reduced in the AGSΔ*cttn* cells as observed in the present study, indicating that the importance of cortactin in the gastric cancer pathway. In conclusion, the diminished activation of both Src and Abl as well as the reduced phosphorylation of CagA point to cortactin as an interesting, potential new biomarker and therapeutic target for the prognosis and treatment of stomach cancer in the future.

## 4. Materials and Methods

### 4.1. Cultivation of Eukaryotic Cells

The human adenocarcinoma cell line AGS (ATCC CRL-1739) was grown at 37 °C and 5% CO_2_ in RPMI 1640 medium (Gibco, Darmstadt, Germany) containing 10% fetal calf serum (Gibco), 1% penicillin/streptomycin (Sigma-Aldrich, Steinheim, Germany), and 0.2% normocin (InvivoGen, Toulouse, France), as described previously [81]. For *H. pylori* infection experiments, the cells were washed twice with PBS buffer to remove all traces of antibiotics and incubated with fresh medium without antibiotics [82]. 

### 4.2. Generation of Cortactin Knockout Cell Lines

To achieve a complete gene knockout of cortactin in AGS cells, the CRISPR/Cas9 system was used (Santa Cruz Biotechnology, Heidelberg, Germany) following a protocol developed in our lab [39]. AGS cells were seeded into a 6-well plate and transfected with 0.8 µg of each of the commercially available CRISPR/Cas9 plasmids sc-400761 (knockout plasmid; Santa Cruz Biotechnology, Heidelberg, Germany) and sc-400761-HDR (HDR-plasmid; Santa Cruz Biotechnology, Heidelberg, Germany). The knockout plasmid induced a double strand break in the *cttn* gene, while the HDR-plasmid inserted a gene-cassette into this break and repaired it. The inserted gene-cassette transfered, among other things, a resistance against the antibiotic puromycin and a fluorescence marker, namely red fluorescent protein (RFP). Transfected cells were selected in media containing 2 µg/mL puromycin, which killed all untransfected cells within 24 h. Afterwards, single-cell-sorting was performed by fluorescence activated cell sorting (FACS Aria II SORP; BD Bioscience, Heidelberg, Germany) using RFP as a signal. Starting in a 96-well plate (Greiner Bio-One, Frickenhausen, Germany), single cell colonies were grown until confluence, and then step-wise transferred into well plates with increasing well sizes. Knockout of cortactin was confirmed in three single cell colonies via Western blotting and immunofluorescence microscopy using a cortactin antibody.

### 4.3. Cultivation of H. pylori Strains

Type1 *H. pylori* wt strains Ka88 [83], N6 [84], G27 [85], P12 [86], and P1 [87] were grown from stocks stored at −80 °C (BHI medium containing 20% glycerol). The bacteria were grown in microaerophilic conditions on GC agar plates with 10% horse serum, 10 µg/mL vancomycin, and 4 µg/mL amphotericin. The microaerophilic conditions were created in a 2.5 L anaerobic jar (Oxoid, Wesel, Germany) with a CampyGen (Oxoid) package. The bacteria were grown from stock for 48 h, then resuspended in BHI medium and incubated on a fresh agar plate for another 18 h before usage in infection experiments. The isogenic P12Δ*cagA* mutant was treated the same as the wild-type strains, except that agar plates additionally contained 8 µg/mL kanamycin for selection.

### 4.4. Infection of AGS wt and AGSΔcttn Cells with H. pylori

AGS wt and AGSΔ*cttn* cells were grown in RPMI medium containing 10% FCS, penicillin/streptomicin, and normocin in 6-well plates to a confluence of approximately 70%. Cells were starved in plain RPMI medium overnight before infection with *H. pylori*. Bacteria were resuspended in BHI from agar plates, as described above. Infections were performed at a multiplicity of infection (MOI) of 100. After infecting AGS cells with the respective bacterial strains, the 6-well plates were incubated at 37 °C and 5% CO_2_ for 6 h before cell harvesting and lysis using hot (95 °C) 1 × SDS buffer [88].

### 4.5. SDS-PAGE and Immunoblot Analysis

Proteins were separated by size using SDS-PAGE with 6–10% polyacrylamide gels, followed by transfer to a PVDF membrane for Western blotting [89]. PVDF membranes were probed with antibodies after being blocked with either 3% BSA, 5% BSA, or 5% non-fat dry milk in TBST according to manufacturers’ instructions. Antibodies used were specific for Cortactin (Merck-Millipore, Darmstadt, Germany; #05-180), Vinculin (Sigma-Aldrich, Steinheim, Germany, #V9131), Talin (Cell Signaling Technology, Frankfurt, Germany, #4021), Paxillin (Santa Cruz Biotechnology, Heidelberg, Germany, sc-5574), FAK (BD Bioscience, Heidelberg, Germany, #610087), Src (Cell Signaling Technology, Frankfurt, Germany, #2123), Abl (Santa Cruz Biotechnology, Heidelberg, Germany, #sc-131), SHP2 (Santa Cruz Biotechnology, Heidelberg, Germany, #sc-7384), α-Actinin (Sigma-Aldrich, Steinheim, Germany, #A5044), CagA (Austral Biologicals, San Ramon, CA, USA; #HPP-5003-9), GAPDH (Santa Cruz Biotechnology, Heidelberg, Germany, #sc-47724), PY99 (Santa Cruz Biotechnology, Heidelberg, Germany, #sc-7020), FAK PY397 (Cell Signaling Technology, Frankfurt, Germany, #3283S), Src PY418 (Oncogene, Heidelberg, Germany, #OP07), and Abl PY412 (Sigma-Aldrich, Steinheim, Germany, #C5240). Secondary antibodies were coupled to horseradish peroxidase and could detect either primary mouse (Invitrogen, Darmstadt, Germany, #31446) or rabbit (Invitrogen, Darmstadt, Germany, #31460) antibodies, and Western blots were developed as described previously [90]. 

### 4.6. Immunofluorescence Microscopy

AGS cells were grown in 10% FCS for 24 h until 50% confluence, followed by fixation in 4% PFA for 10 min at RT. Fixed cells were permeabilized with 0.1% Triton X-100 for 10 min and then immunostained with anti-cortactin (Merck-Millipore, #05-180) mouse IgG followed by secondary, FITC-conjugated goat-anti-mouse antibody. In addition, cells were counterstained with TRITC-conjugated phalloidin (#R415 from Thermo Fisher Scientific, Darmstadt, Germany) for the F-actin cytoskeleton and 4′-6-diamidino-2-phenylindole dihydrochloride (DAPI, from Thermo Fisher Scientific, Darmstadt, Germany) for the detection of cell nuclei. Samples were analyzed by confocal laser scanning microscopy (CLSM) using a Leica SP5 (Leica Microsystems, Wetzlar, Germany) at the facilities of the Optical Imaging Centre Erlangen (OICE, Erlangen, Germany). Excitation/emission wavelengths used for DAPI, FITC, and TRITC fluorophores were 405/413–460 nm, 488/496–550 nm, and 561/571–630 nm, respectively. In addition to immunofluorescence channels, differential interference contrast (DIC) imaging was also applied. The obtained data were visualized using LAS AF computer software (Leica Microsystems). To assess AGS cell areas, the DIC images were analyzed with the ImageJ software [91]. Fluorescence intensities of actin stress fibers were also assessed using the ImageJ software and expressed as relative fluorescence units (RFU), obtained by dividing total cellular fluorescent signal by cellular area. All experiments were performed at least in triplicates. 

### 4.7. Electron Microscopy

Cells were infected as described above and fixed with a solution containing 2% glutaraldehyde and 5% formaldehyde in HEPES buffer (0.1 M HEPES, 0.09 M sucrose, 0.01 M MgCl_2_, 0.01 M CaCl_2_, pH 6.9) for 1 h on ice. After storage at 4 °C, samples were washed twice with TE buffer (TRIS 10 mM, EDTA 2 mM). Then, samples were dehydrated in a graded series of acetone (10%, 30%, 50%, 70%, and 90%) for 30 min each step. The 100% acetone step was carried out twice at room temperature for 10 min. The critical point drying was carried out with the automated system from Leica (CPD 300). Subsequently, samples were sputter-coated with gold/palladium. After mounting on 12 mm aluminium stubs with adhesive tape, samples were observed in a Zeiss Merlin field emission scanning electron microscope at an acceleration voltage of 5 kV and imaged with the inlens-SE detector and Everhart-Thornley SE detector in a 75:25 ratio. 

### 4.8. Transfection of AGSΔcttn Cells

AGSΔ*cttn* cells were transfected with an empty vector control as well as the plasmids pcDNA3-FAK-HA [44], peGFP-N1-Src [45], and pcDNA3-Abl-PP [46], encoding constitutively active versions of FAK, Src, and Abl kinases, respectively, using Turbofect transfection reagent (ThermoFisher Scientific, Waltham, MA, USA) according to manufacturer’s instructions. AGSΔ*cttn* cells were grown to approximately 70% confluence in a 6-well plate with RPMI 1640 medium containing 10% FCS. 5 micrograms of each plasmid were mixed in 200 µL RPMI 1640 plain medium with 10 µL of the turbofect transfection reagent and incubated for 20 min at room temperature. The plasmid/transfection reagent mix was carefully added to each well, followed by incubation of the cells for 24 h at 37 °C and 5% CO_2_ [92]. Transfected cells were subsequently used for *H. pylori* infection experiments as described above. 

### 4.9. Counting of Adherent Bacteria

AGS wt cells were seeded into a 6-well plate and grown to approximately 70% confluence. Afterwards, cells were infected for 6 h with *H. pylori* strain P12 and its isogenic Δ*cagA* mutant as described above at an estimated MOI of 100. Infected cells were washed three times with RPMI1640 medium and then incubated with 1 mL 0.2% saponin buffer (Tris 50 mM; Na_3_VO_4_ 0.4 mM; NaF 1 mM; saponin 0.2%; Complete Proteinase Inhibitor) at 37 °C for 15 min, in order to lyse the eukaryotic cells but not the bacteria. Twenty microliters of lysed cells were diluted in 180 µL BHI medium and plated onto a Mueller-Hinton-plate. The plates were then incubated for 2 days at 37 °C and 5% CO_2_ before the number of colonies, produced by viable *H. pylori*, were counted. 

### 4.10. Statistics

All experiments were done in triplicates. Relative phosphorylation levels of CagA, FAK, Src, and Abl were determined by measuring band intensities densitometrically using Image Lab Software (Version 6.1; Bio-Rad Laboratories, Feldkirchen, Germany). Band intensities or background signals of uninfected cells were set to a value of 1, and fold increase in infected cells determined in comparison. Statistical significance of differences in Western blot signals was evaluated using one-way ANOVA, followed by Tukey’s test with GraphPad Prism statistical software version 8.0 (GraphPad Software, San Diego, CA, USA, www.graphpad.com, accessed on 27 April 2021). Statistical analysis of immunofluorescence microscopy was performed by pair comparison of AGS wt cells to AGSΔ*cttn* clones 1, 4, and 8 employing Mann-Whitney nonparametric two-tailed test. The data obtained in CLSM were analyzed and graphically visualized by using GraphPad Prism 8.0 for Windows (GraphPad Software, San Diego, CA, USA, www.graphpad.com, accessed on 27 April 2021). Statistical significance was defined as * *p* ≤ 0.05, ** *p* ≤ 0.01 and *** *p* ≤ 0.001.

## Data Availability

Data is available upon request from corresponding author.
